# Inhibition of myostatin signaling increases glucose in insulin-deficient diabetic mice

**DOI:** 10.1186/1753-6561-6-S3-P52

**Published:** 2012-06-01

**Authors:** Qian Wang, Tingqing Guo, Alexandra C McPherron

**Affiliations:** 1 Genetics of Development and Disease Branch, NIDDK/NIH, Bethesda, MD, 20852, USA

## Background

Myostatin (MSTN), a TGF-β superfamily member, is a negative regulator of muscle mass that plays an important role in metabolism. *Mstn* KO mice have increased muscle mass, reduced adipose mass and improved insulin sensitivity. We have recently shown that MSTN inhibition in muscle prevents the development of diabetes in a mouse model of lipodystrophy. Whether inhibition of MSTN in a type I diabetes model would improve hyperglycemia is unknown.

## Materials and methods

We used streptozotocin (STZ)-treated C57 mice in which the insulin-producing β-cells were specifically damaged leading to hyperglycemia. After overt diabetes developed, the STZ-treated mice were injected with a MSTN inhibitor, a soluble Activin receptor type II B (ACVR2B:Fc). Blood glucose levels were measured regularly by glucometer. Pyruvate tolerance and glutamine tolerance tests were performed and several hormones in the serum were measured. Real-time PCR was used to compare the expression level of some genes involved in gluconeogenesis.

## Results

The soluble ACVR2B:Fc-treated STZ mice have higher blood glucose levels compared with untreated STZ mice. There were no differences in insulin and glucagon levels between ACVR2B:Fc treated or untreated STZ mice. However, there were higher levels of the glucocorticoid corticosterone in soluble ACVR2B:Fc-treated mice. Real-time PCR data showed that the expression of the *PEPCK* gene was increased significantly in ACVR2B:Fc-treated mice.

## Conclusion

Our data suggest that the soluble ACVR2B:Fc treatment worsens hyperglycemia possibly due to increased gluconeogenesis. These data suggest that MSTN inhibition will not be useful for treating type I diabetes.

